# Adductor Canal Blocks Are Not Associated With Improved Early Postoperative Outcomes in Patients Undergoing Total Knee Arthroplasty

**DOI:** 10.31486/toj.22.0074

**Published:** 2023

**Authors:** Samuel Elliott Holbert, Samantha N. Baxter, Jane C. Brennan, Andrea H. Johnson, Minahil Cheema, Justin J. Turcotte, James H. MacDonald, Paul J. King

**Affiliations:** Department of Orthopedics, Anne Arundel Medical Center, Annapolis, MD

**Keywords:** *Anesthesia–spinal*, *arthroplasty–replacement–knee*, *nerve block*, *postoperative complications*

## Abstract

**Background:** As length of stay after total knee arthroplasty (TKA) continues to shorten, interventions that may reduce early postoperative pain and complications must be studied. Peripheral nerve block is being explored as a potential means of improving pain management. The purpose of this study was to evaluate the impact of adductor canal block (ACB) on postoperative outcomes for patients undergoing TKA.

**Methods:** We conducted a retrospective review of 565 patients who received unilateral TKA under spinal anesthesia with a periarticular anesthetic injection. Patients were divided by ACB status. Univariate comparisons and multivariate regression were used to compare outcomes for patients receiving ACBs vs those who did not.

**Results:** Of the 565 patients, 167 received an ACB, and 398 did not. Patients who received an ACB were less likely to require nausea medication during the immediate postoperative period. Length of stay, narcotic consumption, rate of discharge to home, postanesthesia care unit recovery time, urinary retention, ability to complete physical therapy, and 30-day readmission rate did not differ significantly between groups. After risk adjustment, the only significant finding was decreased likelihood of nausea in patients receiving an ACB.

**Conclusion:** ACBs appear to have little to no significant impact on early clinical outcomes in patients having TKA under spinal anesthesia with a periarticular anesthetic injection. Further study of larger patient cohorts is required to validate these findings.

## INTRODUCTION

Total knee arthroplasty (TKA) is one of the most common procedures performed throughout the world.^1^ Despite the overall success of the procedure, managing early postoperative pain after TKA remains challenging. Approximately 60% of patients report severe pain postsurgery.^2^ Intense postoperative pain can hinder patient recovery and lead to numerous issues, including mobility-related complications such as decreased ability to move the knee, higher risk of deep vein thrombosis, and increased length of hospital stay.^3,4^

As rapid recovery protocols evolve and length of stay continues to decrease after TKA, use of peripheral nerve blockade is being explored as a potential means of improving pain management.^5^ Additionally, anesthesia type has been shown to influence postoperative complication rate and patient satisfaction in elective TKA.^6,7^ Although some studies have shown equivocal outcomes for both anesthesia types, spinal anesthesia has been associated with decreased risk of common surgical complications, such as surgical site infection, in both total hip arthroplasty and TKA patient populations.^7-9^ Despite the benefits of spinal anesthesia, this anesthesia route has also been associated with increased risk of venous thromboembolism.^6,7^ Because of the known impact on surgical outcomes, anesthesia type must be regarded as a possible confounding variable when analyzing the influence of other treatment factors for patients undergoing TKA.^8^

Femoral nerve block (FNB) and adductor canal block (ACB) have been investigated as regional techniques that prolong the effectiveness of anesthesia.^1,2,10^ Historically, the popularity of FNB increased based on its ability to reduce postoperative pain and the need for narcotics after TKA.^11^ However, FNB targets the femoral nerve, affecting both the afferent and efferent nerve pathways and compromising quadriceps muscle strength, which can adversely affect patient mobilization and stabilization postsurgery, and the procedure has been associated with higher instances of complications compared to procedures not utilizing FNBs.^4,12-14^ ACB involves the injection of anesthetic anterolateral to the femoral artery at the mid-thigh in the musculofascial space which anesthetizes the saphenous nerve and the nerve to the vastus medialis while sparing the motor branches of the femoral nerve.^10,15^ The principal strength of this method is the ability to avoid the quadriceps weakness that is associated with FNB and thereby allow for early ambulation after surgery.^16^

The purpose of this study was to add to the current literature by investigating the effect ACB has on early postoperative pain and outcomes after TKA performed under spinal anesthesia with a periarticular anesthetic injection (PAI).

## METHODS

Our institutional clinical research committee deemed this study institutional review board exempt. We conducted a retrospective medical records review of all patients undergoing primary unilateral TKA by 10 board-certified surgeons at a single institution. The timeline for inclusion was February 1, 2020, to September 1, 2020. Patients undergoing bilateral TKA or revision TKA were excluded. Data were collected using an administrative database for patient demographics including age, sex, marital status***,*** body mass index (BMI), and procedure performed. American Society of Anesthesiologists (ASA) physical status classification score 3 or 4 was used to quantify preoperative health status. Intraoperative administration of fentanyl, hydromorphone, tranexamic acid, and dexamethasone was also recorded for each patient.

### Perioperative Protocol

All patients were cared for in a coordinated joint replacement center and received written education materials, preoperative medical evaluations, preoperative home exercise or outpatient physical therapy, and an education class for patients and their caregivers. Based on clinical history, patients received preemptive oral medications including celecoxib, acetaminophen, and pregabalin 2 hours before their procedure. Spinal anesthesia was paired with propofol sedation and consisted of either 12 to 15 mg of hyperbaric or isobaric bupivacaine or 50 to 70 mg of mepivacaine. Patients received intravenous opioids administered intraoperatively and in the postanesthesia care unit (PACU). All patients received a standard PAI of ropivacaine and epinephrine before closure. ACBs were completed using either ropivacaine or bupivacaine administered under ultrasound guidance. The decision to administer an ACB in the operating room prior to incision was made preoperatively in consultation with the patient and anesthesiologist. Postoperatively, all patients were treated according to a multimodal pain management protocol that, depending on patient factors, included acetaminophen, oral nonsteroidal anti-inflammatory drugs, pregabalin, ketorolac, and oral opioid medications as needed. All patients received assisted ambulation on the day of surgery when appropriate.

### Study Outcomes

Postoperative outcomes of interest included PACU nausea and pain, administration of nausea medication within 24 hours of surgery, ability to participate in the first postoperative physical therapy session, urinary retention, narcotic consumption, length of stay (measured in hours), discharge disposition, recovery time (measured in hours), last PACU pain score measured by the 10-point numeric rating scale, and 30-day readmission. Urinary retention was defined as any incidence of recatheterization during the patient's hospital stay. Narcotic consumption was measured as oral morphine milligram equivalents and included all narcotics received outside of the operating room.

### Statistical Analysis

Patients were grouped based on whether they received an ACB at the time of surgery. Statistical analyses were used to determine the impact of ACBs on postoperative outcomes. Univariate analysis, including chi-square tests and 2-sided independent samples *t* tests, was used to determine differences between groups. Fisher exact test was performed when the assumptions of chi-square testing were not met, and the Mann-Whitney *U* test was used for nonparametric continuous data. Multiple logistic and linear regression was used to establish the effect of ACB on postoperative outcomes while controlling for sex, administration of fentanyl, and administration of dexamethasone. These variables were selected as possible confounding factors because they were significantly different at α<0.10 between groups in preliminary univariate analysis. The same control variables were used for all regression models. All statistical analyses were performed using RStudio, version 1.4.1717 (RStudio, Inc.). Statistical significance was assessed at *P*<0.05.

## RESULTS

Of the 565 patients included in the study, 167 (29.6%) received an ACB and 398 (70.4%) did not. No significant differences in age, BMI, marital status, race, or ASA physical status classification score 3 or 4 were seen between patients in the ACB and non-ACB groups (Table 1). A significant difference was found in the distribution of males and females between the 2 groups, with females composing a greater percentage of the ACB group (*P*=0.015). Intraoperatively, the rates of intravenous (IV) tranexamic acid, IV or intrathecal fentanyl, and IV hydromorphone use were similar between the 2 groups (Table 2). However, patients in the ACB group were more likely to receive IV dexamethasone intraoperatively compared to patients in the non-ACB group (*P*<0.001).

**Table 1.** Patient Demographics

	Adductor Canal Block	No Adductor Canal	
Variable	Group, n=167	Block Group, n=398	*P* Value
Age, years, mean ± SD	67.4 ± 8.17	67.7 ± 8.67	0.678
Sex			**0.015**
Female	109 (65.3)	214 (53.8)	
Male	58 (34.7)	184 (46.2)	
Married	104 (62.3)	253(63.6)	0.845
White race	130 (77.8)	333 (83.7)	0.128^a^
Body mass index, kg/m^2^, mean ± SD	30.82 ± 5.12	31.47 ± 5.18	0.175
American Society of Anesthesiologists physical status classification 3 or 4	61 (36.5)	152 (38.2)	0.782

^a^Fisher exact test.

Notes: Data are expressed as n (%) unless otherwise indicated. Significant *P* values are in bold.

**Table 2.** Intraoperative Medications

Medication	Adductor Canal Block Group, n=167	No Adductor Canal Block Group, n=398	*P* Value
Tranexamic acid, IV or topical	149 (89.2)	340 (85.4)	0.284
Dexamethasone, IV	129 (77.2)	201 (50.5)	**<0.001**
Fentanyl, IV or intrathecal	95 (56.9)	257 (64.6)	0.097
Hydromorphone, IV	21 (12.6)	72 (18.1)	0.137

Notes: Data are expressed as n (%). Significant *P* values are in bold.

IV, intravenous.

As shown in Table 3, during the early postoperative period, patients receiving an ACB experienced lower rates of nausea in the PACU (*P*<0.001). However, no significant differences in PACU recovery time, last PACU pain score, first physical therapy failure rate, rate of urinary retention, oral morphine milligram equivalent, or oral morphine milligram equivalent per hour were observed between the 2 groups. Patients in the ACB group experienced a significantly shorter average length of stay than those in the non-ACB group (*P*=0.037). The rate of discharge to home was similar between groups. However, the rate of 30-day readmission was significantly lower among patients in the ACB group (*P*=0.029).

**Table 3.** Hospital and 30-Day Postoperative Outcomes

	Adductor Canal Block	No Adductor Canal	
Outcome	Group, n=167	Block Group, n=398	*P* Value
Nausea in the PACU	30 (18.0)	135 (33.9)	**<0.001**
Inability to complete first physical therapy session^a^	7 (4.2)	32 (8.0)	0.141
Reason: dizziness	6 (85.7)	11 (34.4)	**0.030** ^b^
Reason: orthostasis	3 (42.9)	7 (21.9)	0.344^b^
Reason: pain	3 (42.9)	22 (68.8)	0.225^b^
Urinary retention	8 (4.8)	9 (2.3)	0.182
Length of stay, hours, mean ± SD	29.82 ± 18.48	31.48 ± 17.65	**0.037** ^c^
Oral morphine milligram equivalent, mean ± SD	72.19 ± 47.42	80.19 ± 56.4	0.081
Oral morphine milligram equivalent per hour, mean ± SD	2.63 ± 1.40	2.65 ± 1.34	0.873
Discharge to home	163 (97.6)	384 (96.5)	0.667
30-day readmission	1 (0.6)	16 (4.0)	**0.029** ^b^
PACU recovery time, hours, mean ± SD	157.67 ± 63.93	148.37 ± 51.61	0.097
Last PACU pain score, mean ± SD^d^	2.86 ± 2.11	2.65 ± 2.27	0.283

^a^Some patients who were unable to complete the first physical therapy session failed for multiple reasons, and all the reported reasons for failure were recorded. Therefore, the total number of reasons is greater than the total number of failures reported.

^b^Fisher exact test.

^c^Mann-Whitney *U* test.

^d^The pain numeric rating scale is measured from 0 (no pain) to 10 (worst pain ever experienced).

Notes: Data are expressed as n (%) unless otherwise indicated. Significant *P* values are in bold.

PACU, postanesthesia care unit.

After risk adjustment, we found a decreased likelihood of nausea in the PACU among patients in the ACB group. Patients in the ACB group were 61% less likely to report PACU nausea (odds ratio 0.39, 95% CI, 0.24 to 0.62; *P*<0.001) than patients in the non-ACB group (Table 4). After risk adjustment, no significant differences in urinary retention rate, 30-day readmission rate, inability to complete physical therapy, oral morphine milligram equivalent, length of stay, and PACU recovery time were observed between patients who received an ACB and those who did not (Tables 4 and 5).

**Table 4.** Multivariate Logistic Regression: Adjusted Risk of Postoperative Complications in Patients Receiving Adductor Canal Block

Complication	Odds Ratio	95% CI	*P* Value
Nausea in the PACU	0.39	0.24 to 0.62	**<0.001**
Urinary retention	2.51	0.88 to 7.07	0.079
Inability to complete first physical therapy	0.48	0.19 to 1.07	0.092
30-day readmission	0.13	0.11 to 0.69	0.055

Notes: Data are controlled for sex, intraoperative dexamethasone, and intraoperative fentanyl. Significant *P* values are in bold.

PACU, postanesthesia care unit.

**Table 5.** Multivariate Linear Regression: Adjusted Risk of Postoperative Outcomes in Patients Receiving Adductor Canal Block

Outcome	Estimate (β)	95% CI	*P* Value
Length of stay, hours	–0.65	–3.99 to 2.68	0.700
PACU recovery time, hours	8.21	–2.19 to 18.61	0.121
Oral morphine milligram equivalent	–3.85	–13.52 to 5.81	0.434

Note: Data are controlled for sex, intraoperative dexamethasone, and intraoperative fentanyl.

PACU, postanesthesia care unit.

## DISCUSSION

Previous studies have demonstrated that ACBs are associated with decreased patient-reported pain scores, decreased opioid requirements, and less quadriceps weakness that allows for early mobilization after TKA.^1,2,17,18^ In contrast, our results suggest that ACBs have little impact on early postoperative outcomes for patients undergoing primary unilateral TKA with spinal anesthesia and PAI. We found that patients receiving an ACB had significantly less nausea in the PACU, shorter lengths of stay, and a lower 30-day readmission rate. However, after controlling for possible confounding variables, only nausea in the PACU was significantly reduced by administration of an ACB. All other postoperative complications and outcomes evaluated with regression analysis had similar results between the 2 groups: rate of urinary retention, inability to complete the first physical therapy session, 30-day readmission, length of stay, PACU recovery time, and narcotic consumption. However, significant methodological differences exist between prior studies demonstrating the positive effects of ACB and those of this study. Many early studies demonstrating improved quadriceps strength and early mobility compared ACB with FNB, a procedure that has largely fallen out of favor because of these known drawbacks.^1,2,17^ Further, the studies comparing ACB+PAI with PAI alone, as we did in this study, used a variety of prospective and retrospective designs. In addition, variability exists in the medications used for ACB and PAI, as well as the concomitant analgesics used for pain management.^18^ Based on these limitations and the lack of impact on postoperative outcomes we observed in this study, further evaluation of ACB is required before it is adopted as standard of care.

Patients receiving ACBs in this study were significantly more likely to be female but also more likely to receive dexamethasone, as noted in Table 2. In a retrospective medical records review of 283 patients comparing mobilization distance and length of stay in total joint arthroplasty, female sex was associated with shorter distance of ambulation and increased length of stay.^19^ In comparison, dexamethasone use in anterior total hip arthroplasty was shown to decrease PACU pain, narcotic use, and overall length of stay in a retrospective medical records review of 376 patients.^20^ These 2 variables appear to confound the data in opposite directions, potentially affecting the outcome measurements being evaluated. By using multivariate analysis, our study was better able to control these confounding factors that may have influenced the results.

Other studies have raised similar questions about whether the clinical impact of ACB supports the use of the treatment in patients undergoing TKA.^18,21,22^ Rames et al conducted a retrospective analysis comparing the postoperative outcomes of 624 TKA patients who had a single-shot ACB and a standardized multimodal pain regimen vs 69 patients who only had the multimodal pain regimen.^22^ Patients in the ACB group were able to ambulate 16 feet further during physical therapy and had a 16% higher rate of postoperative day (POD) 1 discharge, while no differences in postoperative narcotic utilization were observed. Based on these findings, the authors decided to limit the use of ACB to select patients known to have preoperative pain or stiffness, given the cost of ACB and the limited clinical impact on perioperative pain control. Similarly, our results suggest limited influence of ACB on postoperative outcomes, including no influence on narcotic requirements. All ACBs in our study were performed prior to incision, while Rames et al included ACBs performed primarily in the PACU.^22^ Despite these methodological differences, the similar results observed in both studies call into question the routine use of ACB in patients undergoing TKA.

In comparison to the results of our study and of Rames et al,^22^ Ma et al and Grosso et al identified similar trends in their evaluations of the combined effect of an ACB with an intraoperative PAI.^18,21^ Ma et al conducted a meta-analysis comparing the effects of ACB+PAI with PAI alone in patients undergoing TKA.^18^ The authors found that ACB+PAI was associated with longer distances walked than PAI alone on POD 1, but they found no differences in pain, narcotic consumption, length of stay, and postoperative complications between groups. Grosso et al conducted a randomized controlled trial of 155 TKA patients who had spinal anesthesia and various intraoperative pain treatments that included ACB alone, PAI alone, or ACB+PAI.^21^ The visual analog scale (VAS) pain score was significantly higher for patients who had ACB alone on POD 1 and POD 3 in comparison to the other 2 groups. In addition, total opioid consumption was higher, and activity level during physical therapy on POD 0 was significantly lower in the ACB alone group.

As part of our standard intraoperative protocol, all patients included in our study received PAI, therefore eliminating our ability to comment on the utility of ACB used in isolation. Similar to the conclusions of both the Grosso et al and Ma et al studies, the addition of ACB to a PAI appeared to have little effect on postoperative outcomes. However, our finding that patients receiving spinal anesthesia with an ACB were just as likely to complete their first physical therapy session, the Grosso et al observation of no difference in steps taken on POD 0 between the PAI and ACB+PAI groups, and the Ma et al finding of significantly longer distances walked on POD 1 in the ACB group provide evidence that the addition of an ACB to PAI does not appear to negatively affect early ambulation.^18,21^ These data are useful to surgeons who are hesitant to incorporate ACBs into their practice based on prior experience with FNBs that reduced patients’ ability to ambulate in the early postoperative period. A meta-analysis and systematic review comparing local infiltrative anesthesia (LIA) to LIA combined with ACB determined that ACB+LIA provides improved pain control, improved range of motion, decreased morphine use on POD 0 and 1, and lower VAS scores on POD 0 and 1.^23^ While our study did not address the use of ACB in isolation, the results from this meta-analysis suggest the methodology used in our study is reflective of current practice patterns.

Although multiple studies have demonstrated limited clinical impact of ACB on perioperative pain control, other studies have provided contradictory evidence that the technique is associated with improved early pain control and outcomes after TKA.^24-26^ Perlas et al retrospectively assessed 298 patients who underwent TKA under spinal anesthesia.^24^ LIA and ACB+LIA were associated with improved early ambulation and a higher rate of being discharged home compared to the previous standard FNB.^24^ Xing et al conducted a meta-analysis of 4 randomized controlled trials and found a correlation between ACB+PAI and reduced pain scores and opioid consumption when compared to PAI alone.^26^ The authors noted a lower incidence of postoperative nausea in patients who had ACB+PAI. Our findings also demonstrated a reduction in the reports of nausea in the PACU for patients who received an ACB. In contrast to the findings presented in Xing et al, ACB+PAI was not associated with improved pain scores or decreased opioid consumption in our patient population.^26^ While previous researchers have compared the clinical impact of ACB+PAI to PAI alone, few studies have directly compared PAI and ACB. Sardana et al conducted a meta-analysis of 6 studies and found a greater reduction in VAS pain scores and opioid consumption for patients receiving PAI compared to patients receiving an ACB.^25^

Given the mixed outcomes of previous studies investigating the impact of ACB in patients undergoing TKA, the cost-effectiveness of the treatment must be evaluated against the possible clinical impact. To evaluate the potential cost impact of ACB, all aspects of the treatment must be considered, including the cost of the medication, physician fee for services rendered, and the time spent administering the block in the operating room (OR). Time optimization in the OR should be a priority, considering that the estimated OR use cost is $36 to $37 per minute.^27^ From a cost-effectiveness standpoint, Bagaria et al evaluated the feasibility of direct ACB as part of surgeon-administered PAI as a replacement for ultrasound-guided ACB by the anesthesiologist.^15^ After consideration of the ability of surgeons to correctly place the analgesic in the adductor canal with direct ACB methods, the authors concluded that direct ACB is a feasible treatment that may reduce the cost of the procedure and OR time for patients undergoing TKA.

Our study has limitations. First, the retrospective observational nature of this work can result in possible selection bias. Additionally, the data included in the analysis were collected from a single institution. Because of the nature of the study, the findings of this work may not be representative of the larger patient population. Despite this limitation, the inclusion of patients who underwent surgery at a single institution does allow for standardized preoperative, perioperative, and postoperative care, which can eliminate some confounding variables that otherwise may have impacted our results. Second, patient ambulation was collected from the physical therapy note in each patient's electronic medical record. Some patients may have ambulated with staff nurses, and those ambulation sessions would not be captured in the data. Future prospective trials randomizing patients to receive spinal anesthesia with and without an ACB within rapid recovery protocols are recommended to validate these findings. Although ACB seems to have had limited clinical impact on the current study cohort, further study regarding specific patient populations who may benefit from this treatment, such as opioid non-naïve patients, is warranted.

## CONCLUSION

Administration of an ACB significantly reduced patient accounts of nausea in the PACU. However, all other postoperative outcomes evaluated in this study were similar regardless of whether a patient did or did not receive an ACB. Despite this minor benefit, ACB seems to be of limited use in this patient population.

## Notes

*This article meets the Accreditation Council for Graduate Medical Education and the American Board of Medical Specialties Maintenance of Certification competencies for Patient Care, Medical Knowledge, and Practice-Based Learning and Improvement.*

## References

[1] Karkhur Y, Mahajan R, Kakralia A, Pandey AP, Kapoor MC. A comparative analysis of femoral nerve block with adductor canal block following total knee arthroplasty: a systematic literature review. J Anaesthesiol Clin Pharmacol. 2018;34(4):433-438. doi: 10.4103/joacp.JOACP_198_18 30774223 PMC6360900

[2] Tan Z, Kang P, Pei F, Shen B, Zhou Z, Yang J. A comparison of adductor canal block and femoral nerve block after total-knee arthroplasty regarding analgesic effect, effectiveness of early rehabilitation, and lateral knee pain relief in the early stage. Medicine (Baltimore). 2018;97(48):e13391. doi: 10.1097/MD.0000000000013391 30508936 PMC6283224

[3] Affas F, Nygårds EB, Stiller CO, Wretenberg P, Olofsson C. Pain control after total knee arthroplasty: a randomized trial comparing local infiltration anesthesia and continuous femoral block. Acta Orthop. 2011;82(4):441-447. doi: 10.3109/17453674.2011.581264 21561303 PMC3237035

[4] Jiang X, Wang QQ, Wu CA, Tian W. Analgesic efficacy of adductor canal block in total knee arthroplasty: a meta-analysis and systematic review. Orthop Surg. 2016;8(3):294-300. doi: 10.1111/os.12268 27627711 PMC5129513

[5] Kirksey MA, Haskins SC, Cheng J, Liu SS. Local anesthetic peripheral nerve block adjuvants for prolongation of analgesia: a systematic qualitative review. PLoS One. 2015;10(9):e0137312. doi: 10.1371/journal.pone.0137312 26355598 PMC4565585

[6] Nakamura M, Kamei M, Bito S, Spinal anesthesia increases the risk of venous thromboembolism in total arthroplasty: secondary analysis of a J-PSVT cohort study on anesthesia. Medicine (Baltimore). 2017;96(18):e6748. doi: 10.1097/MD.0000000000006748 28471968 PMC5419914

[7] Zorrilla-Vaca A, Grant MC, Mathur V, Li J, Wu CL. The impact of neuraxial versus general anesthesia on the incidence of postoperative surgical site infections following knee or hip arthroplasty: a meta-analysis. Reg Anesth Pain Med. 2016;41(5):555-563. doi: 10.1097/AAP.0000000000000437 27380106

[8] Warren J, Sundaram K, Anis H, Spinal anesthesia is associated with decreased complications after total knee and hip arthroplasty. J Am Acad Orthop Surg. 2020;28(5):e213-e221. doi: 10.5435/JAAOS-D-19-00156 31478916

[9] Turcotte JJ, Stone AH, Gilmor RJ, Formica JW, King PJ. The effect of neuraxial anesthesia on postoperative outcomes in total joint arthroplasty with rapid recovery protocols. J Arthroplasty. 2020;35(4):950-954. doi: 10.1016/j.arth.2019.11.037 31883826

[10] Gadsden JC, Sata S, Bullock WM, Kumar AH, Grant SA, Dooley JR. The relative analgesic value of a femoral nerve block versus adductor canal block following total knee arthroplasty: a randomized, controlled, double-blinded study. Korean J Anesthesiol. 2020;73(5):417-424. doi: 10.4097/kja.20269 32842722 PMC7533174

[11] Zhang W, Lin P, Zhang F, Wang J. Femoral nerve block versus obturator nerve block for pain management after total knee replacement: a randomized controlled trial protocol. Medicine (Baltimore). 2020;99(37):e21956. doi: 10.1097/MD.0000000000021956 32925729 PMC7489602

[12] Ilfeld BM, Duke KB, Donohue MC. The association between lower extremity continuous peripheral nerve blocks and patient falls after knee and hip arthroplasty. Anesth Analg. 2010;111(6):1552-1554. doi: 10.1213/ANE.0b013e3181fb9507 20889937 PMC3271722

[13] Bauer M, Wang L, Onibonoje OK, Continuous femoral nerve blocks: decreasing local anesthetic concentration to minimize quadriceps femoris weakness. Anesthesiology. 2012;116(3):665-672. doi: 10.1097/ALN.0b013e3182475c35 22293719 PMC3288409

[14] Kwofie MK, Shastri UD, Gadsden JC, The effects of ultrasound-guided adductor canal block versus femoral nerve block on quadriceps strength and fall risk: a blinded, randomized trial of volunteers. Reg Anesth Pain Med. 2013;38(4):321-325. doi: 10.1097/AAP.0b013e318295df80 23788068

[15] Bagaria V, Kulkarni RV, Valavi A, Choudhury H, Dhamangaonkar A, Sahu D. The feasibility of direct adductor canal block (DACB) as a part of periarticular injection in total knee arthroplasty. Knee Surg Relat Res. 2020;32(1):48. doi: 10.1186/s43019-020-00066-z 32958074 PMC7507271

[16] Borys M, Domagała M, Wencław K, Jarczyńska-Domagała J, Czuczwar M. Continuous femoral nerve block is more effective than continuous adductor canal block for treating pain after total knee arthroplasty: a randomized, double-blind, controlled trial. Medicine (Baltimore). 2019;98(39):e17358. doi: 10.1097/MD.0000000000017358 31574881 PMC6775428

[17] Vora MU, Nicholas TA, Kassel CA, Grant SA. Adductor canal block for knee surgical procedures: review article. J Clin Anesth. 2016;35:295-303. doi: 10.1016/j.jclinane.2016.08.021 27871547

[18] Ma J, Gao F, Sun W, Guo W, Li Z, Wang W. Combined adductor canal block with periarticular infiltration versus periarticular infiltration for analgesia after total knee arthroplasty. Medicine (Baltimore). 2016;95(52):e5701. doi: 10.1097/MD.0000000000005701 28033266 PMC5207562

[19] Gautreau S, Haley R, Gould ON, Canales DD, Mann T, Forsythe ME. Predictors of farther mobilization on day of surgery and shorter length of stay after total joint arthroplasty. Can J Surg. 2020;63(6):E509-E516. doi: 10.1503/cjs.003919 33155976 PMC7747848

[20] Kelly ME, Turcotte JJ, Aja JM, MacDonald JH, King PJ. Impact of dexamethasone on length of stay and early pain control in direct anterior approach total hip arthroplasty with neuraxial anesthesia. J Arthroplasty. 2021;36(3):1009-1012. doi: 10.1016/j.arth.2020.09.015 33012598

[21] Grosso MJ, Murtaugh T, Lakra A, Adductor canal block compared with periarticular bupivacaine injection for total knee arthroplasty: a prospective randomized trial. J Bone Joint Surg Am. 2018;100(13):1141-1146. doi: 10.2106/JBJS.17.01177 29975272

[22] Rames RD, Barrack TN, Barrack RL, Nunley RM. Effect of adductor canal block on acute perioperative pain and function in total knee arthroplasty. J Arthroplasty. 2019;34(7S):S164-S167. doi: 10.1016/j.arth.2019.02.049 30890391

[23] Zuo W, Guo W, Ma J, Cui W. Dose adductor canal block combined with local infiltration analgesia has a synergistic effect than adductor canal block alone in total knee arthroplasty: a meta-analysis and systematic review. J Orthop Surg Res. 2019;14(1):101. doi: 10.1186/s13018-019-1138-5 30971284 PMC6458644

[24] Perlas A, Kirkham KR, Billing R, The impact of analgesic modality on early ambulation following total knee arthroplasty. Reg Anesth Pain Med. 2013;38(4):334-339. doi: 10.1097/AAP.0b013e318296b6a0 23759708

[25] Sardana V, Burzynski JM, Scuderi GR. Adductor canal block or local infiltrate analgesia for pain control after total knee arthroplasty? A systematic review and meta-analysis of randomized controlled trials. J Arthroplasty. 2019;34(1):183-189. doi: 10.1016/j.arth.2018.09.083 30360981

[26] Xing Q, Dai W, Zhao D, Wu J, Huang C, Zhao Y. Adductor canal block with local infiltrative analgesia compared with local infiltrate analgesia for pain control after total knee arthroplasty: a meta-analysis of randomized controlled trials. Medicine (Baltimore). 2017;96(38):e8103. doi: 10.1097/MD.0000000000008103 28930857 PMC5617724

[27] Childers CP, Maggard-Gibbons M. Understanding costs of care in the operating room. JAMA Surg. 2018;153(4):e176233. doi: 10.1001/jamasurg.2017.6233 29490366 PMC5875376

